# Inhibition of intestinal inflammation and fibrosis by *Scutellaria Baicalensis* georgi and *Boswellia serrata* in human epithelial cells and fibroblasts

**DOI:** 10.1002/iid3.70036

**Published:** 2024-10-08

**Authors:** Ilaria Laudadio, Beatrice Leter, Francesca Palone, Salvatore Cucchiara, Claudia Carissimi, Noemi Scafa, Daniela Secci, Roberta Vitali, Laura Stronati

**Affiliations:** ^1^ Department of Molecular Medicine Sapienza University of Rome Viale Regina Elena 324 Rome Italy; ^2^ Department of Maternal Infantile and Urological Sciences Sapienza University of Rome Viale del Policlinico 155 Rome Italy; ^3^ Laboratory of Biomedical Technologies, Italian National Agency for New Technologies, Energy and Sustainable Economic Development (ENEA) Via Anguillarese, 301 Santa Maria di Galeria Rome Italy; ^4^ Department of Chemistry and Pharmaceutical Technologies Sapienza University of Rome P.le Aldo Moro Rome Italy

**Keywords:** *Boswellia Serrata*, fibrosis, IBD, inflammation, *Scutellaria baicalensis* Georgi

## Abstract

**Objective and Rationale:**

Inflammatory bowel disease, including Crohn's disease and ulcerative colitis, manifests with chronic intestinal inflammation and frequent sequential fibrosis. Current pharmacological therapies may show harmful side effects and are not useful for prevention or resolution of fibrosis. Thus, the use of alternative therapies is emerging as a novel useful approach. Previous results suggest that *Scutellaria baicalensis* Georgi (*SBG*) and *Boswellia serrata (BS)* display anti‐inflammatory properties. The aim of this study was to investigate in intestinal epithelial cells and fibroblasts the anti‐inflammatory and anti‐fibrotic potential of *SBG* and *BS*, alone or in combination.

**Methods:**

Human colorectal adenocarcinoma cells (HT29), human intestinal epithelial cells (HIEC6) and human colon fibroblasts (CCD‐18Co) were used. Cells were pretreated with *SBG* and *BS* and then exposed to pro‐inflammatory and pro‐fibrotic cytokines.

**Results:**

*SBG* and *BS* extracts significantly decreased pro‐inflammatory cytokine expression and improved epithelial restitution in HT29 and HIEC6 cells. Besides, fibrotic marker expression, including *SNAIL*, *ACTA2*, *ZNF281*, was strongly reduced. Colon myofibroblasts treated with *SBG* and *BS* showed a significant decrease of fibrotic markers as well.

**Conclusions:**

*SBG* and *BS* extracts significantly reduce inflammation and impair fibrosis in intestinal epithelial cells and colon myofibroblasts. No cooperative effect is observed.

## Graphical abstract

Inflammatory bowel disease manifests with chronic intestinal inflammation, frequently resulting in fibrosis, for which pharmacological therapies are not available. The use of natural bioactive compounds is emerging as a novel promising approach. We showed that Scutellaria baicalensis Georgi and *Boswellia Serrata* extracts significantly reduce inflammation and impair fibrosis in intestinal epithelial cells and colon myofibroblasts.

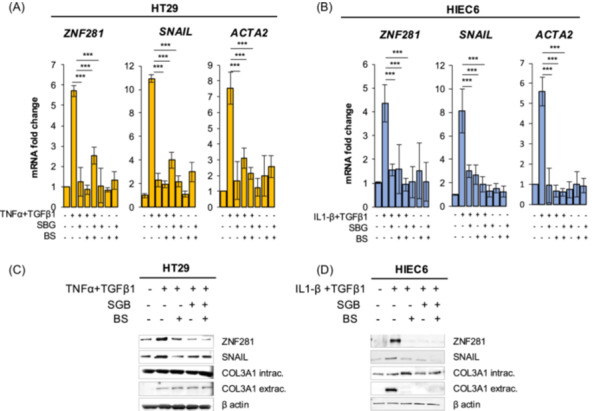

## 1 INTRODUCTION

Inflammatory bowel disease (IBD), which include Crohn's disease (CD) and ulcerative colitis (UC), is a group of chronic intestinal disorders caused by dysregulated immune response in genetically susceptible individuals. The disease is characterized by chronic inflammation of the gastrointestinal wall, leading to increased risk of hospitalization, surgery and disability with a high impact on patients’ quality of life.^1^

Intestinal fibrosis is a common complication of IBD inducing persistent luminal narrowing and strictures, significantly destroying the structure and function of the intestine.^2^ Around 50% of CD patients will develop fibrotic strictures or penetrating lesions, and up to 75% will eventually need surgery.^3^, ^4^ Although less frequent, recent evidence shows some degree of fibrosis in patients with UC as well.^5^, ^6^ The mechanism underlying the development and progression of fibrosis in IBD is still unclear, but growing evidence suggests that chronic intestinal inflammation is an important initiating factor of fibrosis.^7^, ^8^

Currently, traditional pharmacological therapies used in the treatment of IBD involve salicylates, steroids, immunosuppressants or biological agents, that may manifest, in addition to their therapeutic action, harmful side effects, including gastrointestinal disturbance, systemic immunosuppression, kidney toxicity, weight gain, high blood pressure, and increased infections.^9^, ^10^ Besides, the existing anti‐inflammatory drugs do not effectively prevent and relieve fibrosis, so that the incidence of intestinal stricture did not reach a significant drop yet.^11^, ^12^, ^13^, ^14^, ^15^

Newer treatments are now expected to be provided for better efficacy with an improved adverse event profile.

Natural bioactive compounds have already been considered an asset in the therapeutic armamentarium of complex chronic disorders, including digestive tract diseases.^16^, ^17^

*Scutellaria baicalensis* Georgi (*SBG*) is a flowering plant belonging to the Lamiaceae family noted for its high content of bioactive compounds, such as baicalin, baicalein, wogonoside, and wogonin, with high therapeutic potential.^18^ Indeed, anti‐inflammatory,^19^, ^20^ antiviral,^21^ antibacterial,^22^ antioxidant,^23^ anticancer^24^, ^25^ and immunomodulatory^26^ effects of *SBG* have reported.

*Boswellia serrata* (*BS*), belonging to the family of Burseraceae, comprises of a series of pentacyclic triterpene molecules, principally the boswellic acids (BAs), isolated from the gum resin of the plant, that exhibit high efficacy against various chronic diseases such as arthritis, diabetes, asthma, cancer, IBD, Parkinson's disease, Alzheimer's.^21^, ^27^, ^28^ In the resin, more than 12 different boswellic acids have been identified, such as 11‐keto‐β‐boswellic acid (KBA) and 3‐*O*‐acetyl‐11‐keto‐β‐boswellic acid (AKBA), the latter receiving the most significant pharmacological interest.^29^, ^30^

While some evidence has shed light on the anti‐inflammatory properties of *SBG* and *BS*, almost nothing is known about their effects on fibrosis. Thus, the aim of this study was to investigate in vitro, in intestinal epithelial cells and fibroblasts, the anti‐inflammatory and anti‐fibrotic potential of *SBG* and *BS*, alone or in combination.

We show that *SBG* and *BS* extracts significantly reduce inflammation and impair fibrosis in intestinal epithelial cells and colon myofibroblasts. Possibly because of the strong effect displayed by each extract alone, no cooperative effect is observed. Due to the lack of adverse effects, we believe that *SBG* and *BS* extracts may represent very promising candidates for the management of intestinal inflammation and fibrosis.

## 2 MATERIALS AND METHODS

### 2.1 Plant extracts

The dried roots from *SBG* and the dried resin from *BS* were provided by Cadigroup Farmaceutici (Rome, Italy). Dry extracts were obtained by extraction and subsequent evaporation of plants cultivated in China and collected in 2018, according with Good Manufacturing Practice. In *SBG* extracts, baicalin content was certified as 90–95%, as assessed by HPLC. In *BS* extract, boswellic acid contents was 95%.

Dry extracts were weighted and dissolved in ethanol 85% at 1 mg/ml *w*/*v* (stock solution) under sterile conditions and stored at +4°C before using.

### 2.2 Cell lines

All cell lines were purchased from American Type Culture Collection (ATCC, Rockville, MA, USA).

Human colorectal adenocarcinoma cells, HT29, were cultured in McCOY's 5 A medium (Gibco, Life Technologies, Carlsbad, CA, USA), supplemented with 10% heat‐inactivated fetal bovine serum (FBS Eu Approved, Euroclone, Milan, Italy), 2 mM l‐Glutamine, 100 U/ml penicillin and 100 µg/ml streptomycin (Euroclone).

Human intestinal epithelial cells, HIEC6, were cultured in OPTIMEM (Gibco), supplemented with 4% heat‐inactivated FBS, 200 mM HEPES, 10 mM L‐Glutamine (Euroclone) and 10 ng/ml Epidermal Growth Factor (Gibco).

Human colon fibroblasts, CCD‐18Co, were grown in Minimum Essential Medium (MEM, Sigma) supplemented with 10% FBS, 1 mM Sodium Pyruvate, 2 mM l‐Glutamine and Penicillin‐Streptomycin.

### 2.3 Cell viability assay

Cell viability was assessed by MTT assay (Sigma‐Aldrich, Darmstadt, DE) (HT29 and HIEC6) or MTS assay (Promega, Madison, USA) (CCD‐18Co), preformed following manufacturers’ protocols. Briefly, 1.0 × 10^4^ cells were seeded in a 96 multi‐well plate, and the following treatments were performed:

‐ HT29 were pretreated with *SBG* 10 μg/ml and *BS* 10 μg/ml, alone or in combination, in McCOY's 5 A medium complemented with 1% FBS for 24 h (h) and then exposed to 100 ng/ml TNF‐α (Peprotech, Cranbury, USA) and 5 ng/ml TGFβ1 (Abcam, Cambridge, UK) for 48 h;
‐ HIEC6 were pretreated with *SBG* 10 μg/ml and *BS* 10 μg/ml, alone or in combination, in OPTIMEM medium complemented with 1% FBS for 24 h and then exposed to 0.5 ng/ml IL‐1β, (Peprotech, Cranbury, USA) and 10 ng/ml TGFβ1 for 24 h;
‐ CCD18‐Co were pretreated with *SBG* 10 μg/ml and *BS* 10 μg/ml, in MEM medium complemented with 0,5% FBS for 24 h and then exposed to 10 ng/ml TGFβ1 for 24 h.

### 2.4 RT‐qPCR

Total RNA was isolated from cell using the mini RNeasy kit (Qiagen), and 1 μg of total RNA was reverse‐transcribed by IScriptTM cDNA Synthesis Kit (BioRad, Hercules). RT‐PCR amplifications were obtained by a BioRad CFX96 TouchTM Real‐Time PCR Detection System using SsoAdvanced Universal SYBR Green super Mix (BioRad). The following primers were used: ZNF281 (Zinc Finger Protein 281) fwd primer 5′‐GCCATCCTCTCCCCAAGTC‐3′, rev primer 5′‐GAGCTTCGGAAAGCAGCACTA‐3′; SNAIL (Snail Family Transcriptional Repressor 1) fwd primer 5′‐GACCACTATGCCGCGCTCTT‐3′; rev primer 5′‐TCGCTGTAGTTAGGCTTCCGATT‐3′; ACTA2 (Actin Alpha 2, Smooth Muscle) fwd primer 5′‐CCGACCGAATGCAGAAGGA‐3′; rev primer 5′‐ ACAGAGTATTTGCGCTCCGAA‐3′; IL‐8 (Interleukin‐8) fwd primer 5′‐CTGGCCGTGGCTCTCTTG‐3′, rev primer 5′‐CTTGGCAAAACTGCACCTTCA‐3′; IL1B (Interleukin‐1 beta) fwd primer: 5′‐AGACATCACCAAGCTTTTTTGCT‐3′, rev primer: 5′‐GCACGATGCACCTGTACGAT‐3′; TNFA (Tumor Necrosis Factor‐ Alpha) fwd primer 5′‐GGCAGTCAGATCATC‐3′; rev primer 5′‐GCTGCCCCTCAGCTT‐3′; FAP (Fibroblast Activation Protein Alpha) fwd primer 5′‐CCCACGCTCTGAAGACAGAA‐3′; rev primer 5′‐AGTTATGAACTCTTGAAGGGCGT‐3′; FN1 (Fibronectin 1) fwd primer 5′‐AGACCATACCTGCCGAATGTAG‐3′; rev primer 5′‐GAGAGCTTCCTGTCCTGTAGAG‐3′; GAPDH (Glyceraldehyde‐3‐Phosphate Dehydrogenase) fwd primer 5′‐GCACCGTCAAGGCTGAGAAC‐3′ and GAPDH rev primer 5′‐GAGGGATCTCGCTCCTGG‐3′. The expression level of each mRNA was assessed using 2^−ΔΔCt^ method and GADPH was used as housekeeping gene for normalization.

### 2.5 Immunoblot analysis

Cell pellets from HT29, HIEC6 and CCD‐18Co pretreated with *SBG* and *BS* and exposed to pro‐inflammatory and/or pro‐fibrotic cytokines, as described above, were lysed in ice‐cold lysis buffer (50 mM Tris pH 7.4, 5 mM EDTA, 250 mM NaCl, 0.1% Triton X‐100, 1 mM phenylmethylsulfonyl fluoride, 5 mg/ml aprotinin, 5 mg/ml leupeptin, and 1 mM sodium orthovanadate). Ten μg of total proteins were analyzed by western blot using the following antibodies: Anti‐ZNF281 (Abcam, Cambridge, UK), anti‐SNAIL (Cell Signaling, Danvers, MA, USA), anti‐COL3A1 (ThermoFisherScientific, Waltham, MA USA), anti‐β‐actin (Sigma Aldrich), anti‐β‐tubulin (T7816, Sigma).

Culture medium (300 µl) from HT29, HIEC6 and CCD‐18Co was also collected and centrifuged at 2000 r.p.m. for 10 min. Then, supernatants were collected and analyzed by western blot using anti‐COL3A1 and anti‐COL1A1 antibodies (Cell Signaling, Danvers, MA, USA).

### 2.6 Enzyme‐Linked ImmunoSorbent assay (ELISA)

Quantification of human IL‐8, IL‐1β and TNF‐α released in culture medium was carried out by Immunoassay in solid phase ELISA (IL‐8/CXCL8 Quantikine ELISA Kit, IL‐1β/IL1F2 Quantikine ELISA Kit, TNF‐α Quantikine ELISA Kit, R&D System, Minneapolis, MN, USA), according to the manufacture instructions. Positive signals were measured spectrophotometrically at a wavelength of 450 nm by GloMax® Explorer Multimode Microplate Reader (Promega Italia S.r.l., Milan, Italy). IL‐8, IL‐1β and TNF‐α concentration was expressed as pg/ml.

### 2.7 Wound healing assay

HT29 and HIEC6 cells were cultured in 6‐well plated at a density of 4 × 10^5^ cells/ml until confluence reached 90%. A straight‐line wound was made using a 10‐ul pipette tip. Cell debris were removed by a wash with PBS and cells were then maintained in a medium with a reduced percentage of FBS (1%). HT29 and HIEC6 cells were pretreated with *SBG* and *BS*, alone or in combination, then, exposed to to pro‐inflammatory and/or pro‐fibrotic cytokines as described above. Scratch wound closure was visualized under light microscope at day 0 and after 48 h and analyzed by Image J plugin Wound Healing tool. Results were reported as percentage of wound size relative to day 0.

### 2.8 Statistics

All experiments were repeated three times. Data were given as mean ± standard deviation (SD) or standard error mean (SEM). All statistical analyses were carried out using GraphPad Prism 6 software. Comparison among groups was performed using the one‐way ordinary ANOVA test with Welch's correction (significance taken as *p* < .05).

## 3 RESULTS

### 3.1 *SBG* and *BS* extracts significantly decrease pro‐inflammatory cytokine expression in HT29 and HIEC6 cells

To perform experiments, we selected HT29 cells with epithelial morphology; moreover, as a more physiological model to investigate effects of plant extracts on inflammation and on inflammation‐derived fibrosis, we also used the non‐tumorigenic intestinal epithelial cells HIEC6.

**Figure 1:**
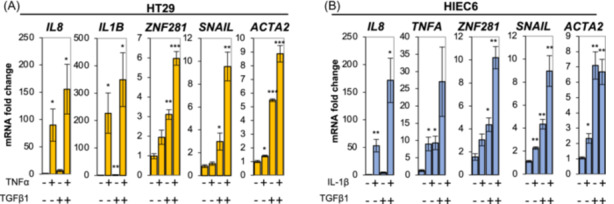
Co‐treatment with pro‐inflammatory and pro‐fibrotic cytokines induces an inflammatory‐driven fibrosis in intestinal epithelial cell lines. HT29 cells were treated with the pro‐inflammatory cytokine TNF‐α (100 ng/ml) and the pro‐fibrotic cytokine TGFβ1 (5 ng/ml) for 48 h (A). HIEC cells were treated with the pro‐inflammatory cytokine IL‐1β (0.5 ng/ml) and TGFβ1 (10 ng/ml) for 24 h (B). The expression of inflammatory (*IL8*, *IL1B*, and *TNFA*), mesenchymal (*ZNF281* and *SNAIL*), and fibrotic (*ACTA2*) markers was assessed by RT‐qPCR. Data are expressed as mean ± SEM. * = *p*‐value ≤ .05; ** = *p*‐value ≤ .01 *n* = 3.

Firstly, we set up a protocol to induce an inflammatory‐driven fibrosis.^8^, ^31^ Intestinal epithelial cells were treated with the pro‐inflammatory cytokine TNF‐α and the pro‐fibrotic cytokine TGFβ1 for 48 h (HT29) or with IL‐1β and TGFβ1 for 24 h (HIEC6). As assessed by RT‐qPCR, treatments increased the expression levels of inflammatory (*IL8*, *IL1B* namely IL‐1β, *TNFA* namely TNF‐α), mesenchymal (*ZNF281* and *SNAIL*), and fibrotic (*ACTA2*, also known as αSMA, α‐smooth muscle actin) markers in both cell lines (Figure 1).

**Figure 2:**
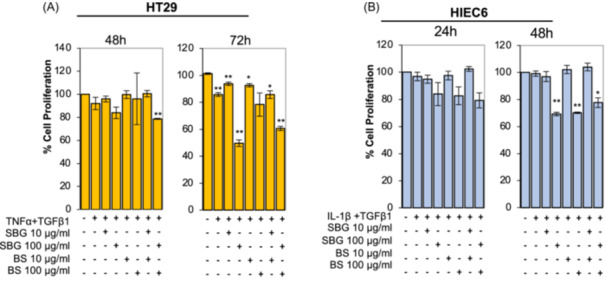
*SBG* and *BS* extracts do not affect viability of intestinal epithelial cells. HT29 cells were pretreated with vehicle (Et‐OH 85%) or with *SBG* (10 μg/ml) and/or *BS* (10 μg/ml) extracts for 24 h and then exposed to the pro‐inflammatory cytokine TNF‐α (100 ng/ml) and pro‐fibrotic cytokine TGFβ1(5 ng/ml) for 48 h (A). HIEC cells were pretreated with vehicle (Et‐OH 85%) or with *SBG* (10 μg/ml) and/or *BS* (10 μg/ml) extracts for 24 h and then exposed to the pro‐inflammatory cytokine IL‐1β (0.5 ng/ml) and pro‐fibrotic cytokine TGFβ1(10 ng/ml) for 24 h (B). Cell viability was assessed by MTT assay. Data are expressed as mean ± SEM. * = *p*‐value ≤ .05; *** = *p*‐value ≤ .001 *n* = 3.

Then, cells were pretreated with vehicle (Et‐OH 85%), *SBG* and *BS* extracts for 24 h and exposed to TNF‐α and TGFβ1 (HT29) or to IL‐1β and TGFβ1 (HIEC6). First, we analyzed the impact of two different concentrations of *SBG* and *BS* extracts on cell viability. Results showed that extracts from both plants, alone or in combination, did not affect HT29 or HIEC6 cell viability after 48 h (HT29) or 24 h (HIEC6), while the higher concentration reduced cell viability after 72 h (HT29) or 48 h (HIEC6) (Figure 2).

**Figure 3:**
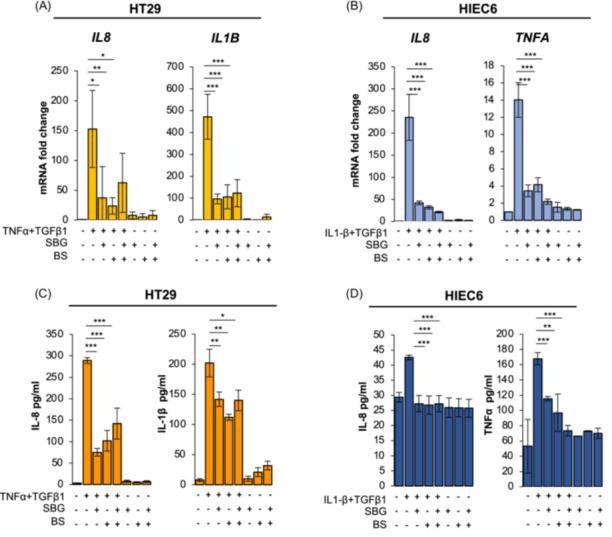
*SBG* and *BS* extracts downregulate the expression and the secretion of pro‐inflammatory cytokines in intestinal epithelial cells. HT29 cells were pretreated with vehicle, *SBG* (10 μg/ml) and *BS* (10 μg/ml) extracts for 24 h and then exposed to TNF‐α (100 ng/ml) + TGFβ1(5 ng/ml) for 48 h. Expression of *IL8* and *IL1B* was assessed by RT‐qPCR (A) Secretion of IL‐8 and IL‐1β (C) was assessed by ELISA. HIEC6 cells were pretreated with vehicle, *SBG* (10 μg/ml) and *BS* (10 μg/ml) extracts for 24 h and then exposed to IL‐1β (0.5 ng/ml) + TGFβ1(10 ng/ml) for 24 h (B). Expression of *IL8* and *TNFA* was assessed by RT‐qPCR (B) Secretion of IL‐8 and TNF‐α was assessed by ELISA (D). Data are expressed as mean ± SEM. * = *p*‐value ≤ .05; ** = *p*‐value ≤ .01; *** = *p*‐value ≤ .001 *n* = 3.

Thus, we selected the lowest concentration for 48 h (HT29) and 24 h (HIEC6) for following experiments. Then, we analyzed the mRNA expression of pro‐inflammatory cytokines (*IL8* and *IL1B* for HT29, *IL8* and *TNFA* for HIEC6). The exposure to TNF‐α + TGFβ1 (HT29) or IL‐1β + TGFβ1 (HIEC6) upregulated the expression of pro‐inflammatory cytokines, however, it was significantly reduced by the treatment with *SBG* and *BS* extracts, alone or in combination (Figure 3A,B).

Further, in HT29 and HIEC6 cells secretion of pro‐inflammatory cytokines upon inflammatory/fibrotic insult was significantly decreased by *SBG* and *BS* as well (Figure 3C,D).

### 3.2 *SBG* and *BS* extracts improve epithelial restitution during inflammation in HT29 and HIEC6 cells

To analyze the effect of *SBG* and *BS* on wound healing process after inflammatory/fibrotic injury, confluent HT29 cells were scratched with a 10 µl micropipette tip and the gap widths (1 mm at day 0) were measured after 48 h, when 50% physiological wound healing occurred in these cells.

**Figure 4:**
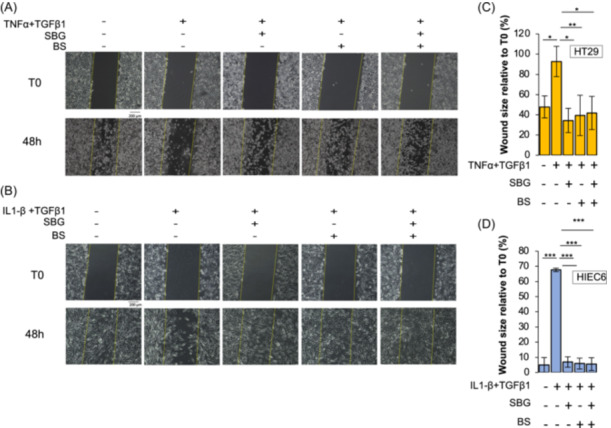
*SBG* and *BS* extracts improve epithelial restitution in intestinal epithelial cells exposed to inflammatory and fibrotic stimuli. Scratch test was performed in HT29 (A) and HIEC6 (B) cells pretreated with vehicle, *SBG* (10 μg/ml) and *BS* (10 μg/ml) extracts for 24 h and then exposed to TNF‐α (100 ng/ml) + TGFβ1(5 ng/ml) or to IL‐1β (0.5 ng/ml) + TGFβ1(10 ng/ml), respectively, for 48 h. Bar charts of cellular density of wounded area in HT29 (C) and HIEC6 (D) relative to T0 are depicted. Data are expressed as mean ± SD. * = *p*‐value ≤ .05; ** = *p*‐value ≤ .01; *** = *p*‐value ≤ .001 *n* = 3.

Scratched cells were exposed to TNF‐α + TGFβ1 or pretreated with vehicle, *SBG* and *BS* extracts for 24 h and then exposed to TNF‐α + TGFβ1 for 48 h. Results showed that cells exposed to inflammatory/fibrotic agents showed a delay of healing of about 45% after 48 h as compared to control cells. However, pretreatment with *SBG* and/or *BS* significantly improved the rate of epithelial restitution, reaching values comparable to control. No cooperative effect between plant extracts was observed (Figure 4A,C).

These data were validated in HIEC6 cells. Again, co‐treatment with *SBG* or *BS* significantly improved the rate of epithelial restitution and no cooperative effect was observed (Figure 4B,D).

### 3.3 Fibrotic marker expression is strongly reduced in intestinal epithelial cells exposed to *SBG* and *BS* extracts

**Figure 5:**
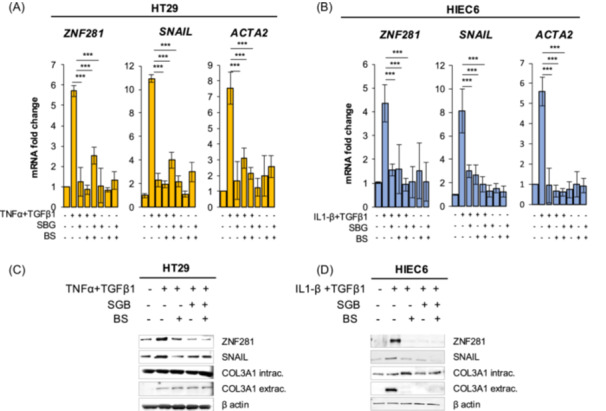
Fibrotic marker mRNAs and proteins are significantly reduced by *SBG* and *BS* extracts in intestinal epithelial cells exposed to inflammatory and fibrotic stimuli. HT29 cells were pretreated with vehicle, *SBG* (10 μg/ml) and *BS* (10 μg/ml) extracts for 24 h and then exposed to TNF‐α (100 ng/ml) + TGFβ1(5 ng/ml) for 48 h. Expression of *ZNF281*, *SNAIL* and *ACTA2* was assessed by RT‐qPCR (A). HIEC6 cells were pretreated with vehicle, SBG (10 μg/ml) and BS (10 μg/ml) extracts for 24 h and then exposed to IL‐1β (0.5 ng/ml) + TGFβ1(10 ng/ml) for 24 h. Expression of *ZNF281*, *SNAIL* and *ACTA2* was assessed by RT‐qPCR (B). Data are expressed as mean ± SEM. * = *p*‐value ≤ .05; ** = *p*‐value ≤ .01; *** = *p*‐value ≤ .001 *n* = 3. ZNF281 and SNAIL protein expression was analyzed by western blot in cell lysates and COL3A1 secretion in cell supernatant (C, D).

Intestinal epithelial cells have been closely involved in fibrosis due to the occurrence of the epithelial‐to‐mesenchymal transition (EMT) process that is critical for cellular conversion from epithelial to mesenchymal phenotypes. Thus, HT29 and HIEC6 were pretreated with *SBG* and *BS* extracts for 24 h and then exposed to TNF‐α + TGFβ1 (HT29) or IL‐1β + TGFβ1 (HIEC6) for 24 and 48 h, respectively. To assess the putative role of plant extracts in controlling fibrosis, fibrotic markers including *SNAIL*, *ACTA2*, collagen type 3 alpha 1 chain (*COL3A1*) and *ZNF281* were analyzed. Results showed that *SNAIL*, *ACTA2* and *ZNF281* mRNA expression, that was increased by the mix of inflammatory/fibrotic cytokines treatment, was significantly reduced in all cells after the treatment with *SBG* and *BS* extracts, alone or in combination (Figure 5A,B).

Accordingly, protein expression of SNAIL, and ZNF81 was importantly decreased, while the co‐exposure did not show further effect. Similarly, the amount of secreted COL3A1, analyzed in cell supernatants, was significantly reduced by the pretreatment with *SBG* and *BS* extracts (Figure 5C,D).

### 3.4 Fibrosis is toughly reduced in colon myofibroblasts exposed to *SBG* and *BS* extracts

**Figure 6:**
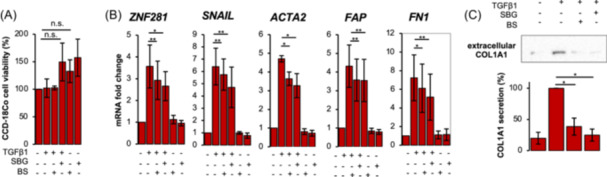
Fibrotic markers are reduced by *SBG* and *BS* extracts in intestinal fibroblasts exposed to fibrotic stimulus. CCD18‐Co cells were pretreated with *SBG* (10 μg/ml) and *BS* (10 μg/ml) for 24 h and then exposed to 10 ng/ml TGFβ1 for 24 h. Cell viability was assessed by MTS assay (A). Expression of *ZNF281*, *SNAIL*, *ACTA2*, *FAP* and *FN1* was assessed by RT‐qPCR (B). Data are expressed as mean ± SEM. * = *p*‐value ≤ .05; ** = *p*‐value ≤ .01; *** = *p*‐value ≤ .001 *n* = 4. CCD18‐Co were pretreated with *SBG* (10 μg/ml) and *BS* (10 μg/ml) for 24 h and then exposed to 10 ng/ml TGFβ1 for 24 h. COL1A1 secretion was analyzed in cell supernatants by western blot and normalized on total secreted proteins. Densitometric analysis was performed (C). * = *p*‐value ≤ .05; *n* = 3.

Myofibroblasts, belonging to the mesenchymal repertoire, are the major cell type involved in fibrogenesis due to their ability in secreting extracellular matrix (ECM) components. Thus, colonic myofibroblasts CCD‐18Co were used to strengthen previous results. Hence, cells were pretreated with *SBG* or *BS* extracts for 24 h and then exposed to the pro‐fibrotic cytokine TGFβ1 for 24 h. As in epithelial cells, the exposure to plant extracts, at the same concentration as before, did not affect cell viability of myofibroblasts (Figure 6A). Analysis of mRNA expression of the fibrotic markers *SNAIL*, *ACTA2*, Fibroblast activation protein (*FAP*), Fibronectin (*FN1*) and *ZNF281*, known to be upregulated during fibrosis in colon fibroblasts,^32^ showed a significant decrease after the exposure to *SBG* or *BS* extracts (Figure 6B).

Likewise, the secreted COL1A1 returned to control value following the extract exposure (Figure 6C).

## 4 DISCUSSION

According to recent evidence, the use of natural compounds as alternative therapies in chronic inflammatory autoimmune diseases, has emerged as a promising approach in gastrointestinal diseases.^33^ Currently, the consumption of complementary and alternative medicines, such as the herbal therapy, is becoming more popular among IBD patients.^34^, ^35^, ^36^, ^37^, ^38^ In this study, we demonstrate the anti‐inflammatory and anti‐fibrotic effects of *SBG* and *BS* on human intestinal cells, suggesting that these promising herbal drugs deserve be considered for IBD management in the future.

First, the anti‐inflammatory properties of *SBG* and *BS* extracts were analyzed. We used intestinal epithelial cells that were previously exposed to plant extracts followed by inflammatory/fibrotic agent treatment. After 24–48 h, we observed a significant reduction of pro‐inflammatory cytokine levels in cells treated with *SBG* or *BS*. No synergistic effect was seen, probably due to the strong effect of each extract alone. Moreover, much evidence supports the view that the maintenance of a healthy intestinal barrier is of crucial importance, indeed, gastrointestinal disorders, including IBD, are often strictly correlated with barrier dysfunctions.^39^, ^40^ The loss of barrier integrity is normally restored through wound healing, a highly complex and coordinated process resulting in tissue restitution. When inflamed, cells undergo a delay in healing. Interestingly, in this study, intestinal epithelial cells pretreated with *SBG* or *BS* extracts significantly improved tissue repair as compared to the positive control. Again, no cooperative effect between *SBG* and *BS* extracts was observed.

All these results highlight an evident role of plant extracts in reducing intestinal inflammation. This evidence is in agreement with previous studies showing that *SBG* and *BS* extracts show anti‐inflammatory effects and attenuate tissue damage in mice with experimental colitis.^41^, ^42^, ^43^, ^44^, ^45^, ^46^

Developing antifibrotics from natural products may represent a remarkable strategy because it reduces the occurrence of adverse collateral effects due to toxicity and improves the safety profile, while maintaining the therapeutic effectiveness, as previously shown in the context of hepatic and pulmonary fibrosis.^47^, ^48^

Previously, the study of Latella et al. suggested that the administration of *SBG* and *BS* was effective in preventing colon fibrosis in rats with a TNBS‐induced colitis.^49^ However, results are still insufficient and further evidence is mandatory to consider these compounds represent reliable tools for the prevention and/or resolution of fibrosis.

First, we used intestinal epithelial cells for experimentation since it is known that during fibrosis they may undergo to the EMT process changing phenotype with loss of epithelial features and acquisition of mesenchymal characteristics and contributing to the ECM deposition.^50^ Previous literature showed that damaged epithelial cells may act as crucial sources of fibroblasts contributing to organ fibrosis through EMT in IBD.^51^ Accordingly, our results showed that in both cell lines, in which fibrosis was induced, as evidenced by the increased levels of fibrotic markers, the treatment with plant extracts caused a significant drop of the expression of the fibrotic markers αSMA and SNAIL, as well as a decrease of COL3A1 release. We also analyzed the expression level of ZNF281, a protein traditionally characterized as an EMT‐inducing transcription factor, but that we have recently found to play a role in gut fibrosis as a novel regulator of colon fibroblast activation and myofibroblast differentiation.^32^ Accordingly, ZNF281 was increased during fibrosis and was strongly reduced by the plant extract exposure as well.

We then analyzed the putative antifibrotic effect of *SBG* and *BS* in myofibroblasts from colonic mucosa (CCD‐18Co). Indeed, in the process of intestinal fibrosis, myofibroblasts are considered the pivotal effectors as appropriately programmed to secrete excessive amount of ECM components. Accordingly, in IBD patients, stromal cells, including pre‐existing myofibroblasts activated by inflammatory stimulants proliferate as a response to inflammation.^52^ CCD‐18Co cells were pretreated with *SBG* and *BS* extracts and induced to develop fibrosis. We observed that myofibroblasts significantly increased the expression levels of fibrotic markers, SNAIL, αSMA, FAP, FN1 and ZNF281, that were likewise downregulated by the exposure to *SBG* or *BS* extracts. Moreover, the level of secreted COLA1 was also significantly reduced.

All these findings clearly demonstrate that *SBG* or *BS* extracts show anti‐fibrotic properties in different intestinal cell types. It is worth noting that *SBG* and *BS* are very effective in impairing fibrosis when administered alone, however, the combination of both has no further effect and no synergism has been revealed.

IBD causes relapsing gut inflammation with the common occurrence of serious complications, especially fibrosis, that result in a severe loss of patients’ quality of life. To introduce a new generation of treating regimen for managing inflammation and inflammatory‐derived fibrosis in IBD with fewer critical side effects, we have explored promising candidates from natural resources. This study shows that *SBG* and *BS* extracts significantly reduce inflammation in intestinal epithelial cells. More interestingly, they also display an evident anti‐fibrotic effect in both intestinal epithelial cells and myofibroblasts. Although actually based on in vitro models, this findings are particularly relevant since there are still very limited treatment options available for managing gut fibrosis. This limitation will be addressed in future research that will include in vivo models. Long‐term studies are necessary before *SBG* and *BS* can be translated from bench to bedside, however, we believe that this study will greatly contribute to elucidate the therapeutic potential of *SBG* to *BS* in IBD.

## AUTHOR CONTRIBUTIONS

Ilaria Laudadio: Conceptualization; Investigation; Supervision; Writing—original draft; Writing—review and editing. Beatrice Leter: Investigation. Francesca Palone: Investigation. Salvatore Cucchiara: Data curation. Claudia Carissimi: Validation. Noemi Scafa: Validation. Daniela Secci: Methodology. Roberta Vitali: Methodology. Laura Stronati: Conceptualization; Funding acquisition; Supervision; Writing—original draft; Writing—review and editing.

## CONFLICT OF INTEREST STATEMENT

The authors declare no conflicts of interest.

## Data Availability

The data that support the findings of this study are available from the corresponding author upon reasonable request.
